# It can be safe to discontinue oral anticoagulants after successful atrial fibrillation ablation: A systematic review and meta-analysis of cohort studies

**DOI:** 10.1097/MD.0000000000035518

**Published:** 2023-10-20

**Authors:** Xiangyu Wang, Minghua Li, Xishu Wang, Zhiguo Zhang

**Affiliations:** a Department of Cardiology, The First Hospital of Jilin University, Changchun, Jilin Province, China.

**Keywords:** atrial fibrillation, blanking period, catheter ablation, major bleeding events, meta-analysis, oral anticoagulants, thromboembolism

## Abstract

**Background:**

Current guidelines recommended that oral anticoagulants (OACs) should last for a minimum first 2 months after atrial fibrillation (AF) ablation and the long-term decision of anticoagulation after AF ablation should be based on the individual patient’s risk of stroke rather than the rhythm status. There is controversy about the safety of discontinuing OACs in patients with atrial fibrillation after the blanking period due to the divergences between consensus recommendations and clinical practice.

**Methods:**

Electronic bibliographic sources (PubMed, Embase, and Web of Science) were searched until August 2023 to identify cohort studies about the safety of discontinuing OACs in patients with AF after the blanking period. The primary outcome was thromboembolism (TE). The secondary outcome was major bleeding events (MBEs). Two authors extracted articles independently using predefined data fields. The pooled odds ratios (ORs) and 95% confidence intervals (CIs) were calculated based on a random-effects model.

**Results:**

A total of 16 studies (11 prospective cohorts and 5 retrospective cohorts) enrolling 23,942 patients (14,382 OFF-OAC and 9560 ON-OAC) were included in our analysis. No significant difference emerged in the risk of TE between OFF-OAC and ON-OAC patients following AF ablation after the banking period (OR = 0.66; 95%CI, 0.43–1.01). Similar results emerged in the patients with a high risk of TE after stratification by the risk level of TE (OR = 0.72; 95%CI, 0.25–2.08). A significant reduction in incidences of major bleeding was found in the OFF-OAC patients compared with the ON-OAC patients (OR = 0.23; 95%CI, 0.12–0.42). Subgroup analyses for TE found a reduction of incidences in the subgroups who switched to antiplatelet drugs and with a follow-up duration <3 years. Subgroup analyses for MBEs found a significant reduction of incidences in all subgroups.

**Conclusions:**

Our study suggests it can be safe to discontinue OACs after successful AF ablation. Discontinuation of OACs may reduce the risk of MBEs while not increasing the risk of TE.

## 1. Introduction

Atrial fibrillation (AF) is a common cardiac arrhythmia that increases the risk of stroke, disability, and mortality.^[1–4]^ Anticoagulation therapy has been the basis for preventing stroke and reducing the occurrence of thromboembolism events. Oral anticoagulants (OACs) are extensively used in patients with high stroke risk, however, OACs carry an increased risk of major bleeding events (MBE), which are often life-threatening.^[4]^ Catheter ablation has been the preferred first-line therapy for atrial fibrillation because it reduces the risk of AF recurrences and AF burden more effectively than antiarrhythmics.^[4,5]^ Despite the benefit of catheter ablation, it is still controversial whether catheter ablation reduces the incidence of stroke and the best anticoagulation therapy strategy after catheter ablation has not been determined.^[5–7]^ The current guideline recommended OACs should be maintained for at least 2 months and an individual patient’s risk of stroke should be taken into account when deciding whether to continue OACs, which is judged by the use of validated risk scores (i.e., CHADS_2_ score or CHA_2_DS_2_-VASc score) regardless of subsequent maintenance of sinus rhythm.^[4]^ However, discontinuation of anticoagulants is very common in AF post-ablation patients despite clear guidelines recommendations according to our clinical experience and literature review and so far no randomized controlled trial (RCT) data can show whether it is safe to discontinue oral anticoagulants. Registries and electronic health records suggest that OACs discontinuations are common in the real world.^[8]^ Thus we conducted a systematic review and meta-analysis to evaluate the safety of discontinuing OACs after the blanking period of successful AF ablation.

## 2. Methods

Our meta-analysis of cohort studies was carried out following Preferred Reporting Items for Systematic Reviews and Meta-Analyses (PRISMA) guidelines.^[9]^ All analyses were based on previous published studies, thus no ethical approval and patient consent are required.

### 2.1. Literature search

Studies were identified by searching the electronic databases of PubMed, Embase, and Web of Science (from inception to August 2023) with a search strategy of combined terms, including “atrial fibrillation,” “ablation,” “anticoagulant,” “non-vitamin K antagonist oral anticoagulant,” and “Warfarin.” No language restriction was applied. Table S1, Supplemental Digital Content, http://links.lww.com/MD/K186 provides the details of the search strategy. References of the relevant review were hand-searching to ensure efficiency.

### 2.2. Study selection

Studies fulfilled the following criteria were included: (1) cohort study; (2) reporting incidence of TE events, including stroke and transient ischemic attack (TIA) and/or incidence of MBEs; (3) patients after successful AF catheter ablation and were divided into OFF-OAC (discontinue OACs after the blanking period) and ON-OAC (continue OACs after the blanking period); (4) follow-up duration lasted for at least 12 months; (5) patients after AF ablation continued OACs at least 3 months. Articles were excluded if they met at least 1 following criteria: (1) not targeted article types (reviews, abstracts, letters, comments, or conference abstracts); (2) patients accepted surgical ablation of left atrial appendage occlusion; (3) not reported targeted outcomes; (4) data provided had significant errors; (5) data cannot be extracted. Disagreements were resolved by discussion. If 2 authors cannot reach an agreement, a third reviewer made the final decision.

### 2.3. Data extraction

Standardized Excel files were designed and were used to extract data from all included sources by 2 authors independently. The following information was extracted: (1) first author, year, country, characteristics of participants at baseline, follow-up duration, type of AF, ablation energy, type of OAC, time of OAC discontinuation, whether converted to an antiplatelet drug, AF monitoring, the definition of AF recurrence, reinitialization of OAC after AF recurrence, blanking period duration; (2) primary outcomes (TE events) and secondary outcomes (MBEs), only interested in late events which defined as events occurred after blanking period; (3) CHADS_2_ and/or CHA_2_DS_2_-VASc scores.

### 2.4. Statistical analysis

Our primary outcomes are risks of TEs and MBEs in patients after successful AF catheter ablation. The odds ratios (ORs) were used to determine the outcomes and the pooled results. We used the Mantel-Haenszel method with random-effects modeling to obtain pooled ORs and 95% confidence intervals (CIs). A 2-tailed *P* < .05 indicates statistically significant. In this study, we used the *I*_2_ index to assess heterogeneity. A high degree of heterogeneity was defined as *I*^2^ > 75%, and a low degree of heterogeneity as *I*^2^ < 25%.^[10]^ Analyses of sensitivity were conducted by eliminating each included study one by one to make sure the results remained robust. Subgroups of patients were analyzed based on their characteristics to examine the potential causes of heterogeneity. Publication bias was quantified utilizing Begg test or Egger test. STATA (version 15.0) and Review Manager (version 5.4) were both used for statistical analysis.

### 2.5. Quality assessment

A Newcastle-Ottawa Scale score was calculated by 2 authors independently regarding the quality of the articles included in this study.^[11]^ There are 3 criteria for evaluating cohort studies: selection (0–4 points), comparability (0–2 points), and outcome evaluation (0–3 points). A total score of at least 8 points indicates high quality, and a 5-to-seven-point score indicates moderate quality. Disagreements were settled through discussion.

## 3. Results

### 3.1. Search results

**Figure 1.**
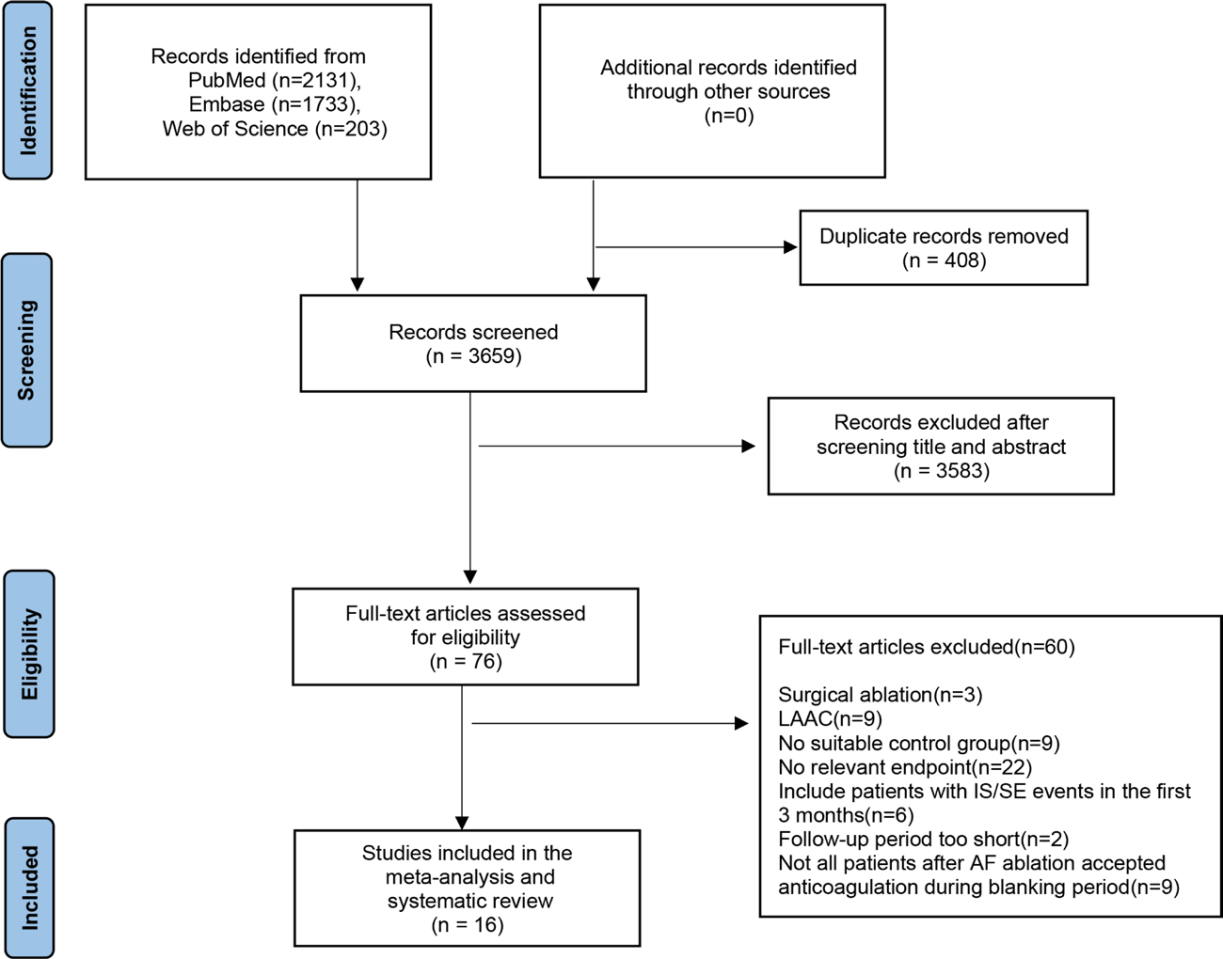
Study selection according to the PRISMA model. PRISMA: the preferred reporting items for systematic reviews and meta-analyses.

A total of 4067 items were found in our search, and 408 duplicates were excluded. After screening titles and abstracts, 76 references were deemed to meet the inclusion criteria. No retrieved potential studies met our inclusion criteria. In total, 16 studies were considered after browsing full texts.^[12–27]^ Figure 1 shows the detailed study flow diagram.

### 3.2. Study characteristics and quality evaluation

Among the studies that were included, 11 were prospective cohort studies^[12,13,15–18,20,21,25–27]^ and 5 were retrospective cohort studies.^[14,19,22–24]^ Among these studies, 4 studies^[15,24,26,27]^ were conducted in Asian countries and others were in non-Asian countries. The duration of follow-up of included studies was at least 12 months. Warfarin was the only anticoagulant in 10 studies,^[12–19,21,22]^ and in 6 studies patients were on warfarin or non-vitamin K antagonist oral anticoagulants (NOACs).^[20,23–27]^ In all studies, OACs were replaced by antiplatelet drugs in the majority of discontinuation groups but 4 studies.^[17,24,25,27]^ The main reasons for stopping OACs were a lower stroke risk and the persistence of sinus rhythm, and the main reasons for continuing OACs were arrhythmic recurrences and left atrium dysfunction. Among 16 studies, stroke events were reported in both groups of patients, and MBEs were reported in both groups of patients in 13 studies.^[12–16,18–22,24,26,27]^ A total of 23,942 patients were included in the studies, consisting of 14,382 patients who were off OACs and 9560 patients who were on OACs. All patients in the OFF-OAC group discontinued OACs after at least 3 months. The average age ranged from 51 to 66 years in patients off OACs and 58 to 66 years in those on OACs. There were 62% to 88% of males in patients off OACs and 55% to 79% in those on OACs. The average CHADS_2_ score and CHA_2_DS_2_-VASc score ranged from 0.4 to 1.06 and 0.7 to 2.3 in patients off OACs and from 0.63 to 1.5 and 1.8 to 2.7 in patients on OACs respectively. Using the NOS score, 12 studies^[12–14,16,17,19,21,22,24–27]^ were assessed to be high quality while 4^[15,18,20,23]^ were considered moderate quality. Baseline characteristics and results of quality assessment of the studies are shown in Table 1.

**Table 1:** Baseline characteristics of studies included in the meta-analysis.

Author and year (country)	Study design	Type of OAC	Sample size	Follow-up (years)	Time of OAC discontinuation (months)	Reinitialization of OAC after AF recurrence	CHA_2_DS_2_ (CHA_2_DS_2_-VASc) score OFF-OAC	CHA_2_DS_2_ (CHA_2_DS_2_-VASc) score ON-OAC	NOS
Oral et al, 2006^[12]^(USA)	Prospective	Warfarin	755	2.1 ± 0.7	3	Unclear	0 = 53%, ≥1 = 47% (0.47†)	0 = 37%, ≥1 = 63% (0.63†)	9
Nademanee et al, 2008^[13]^(USA)	Prospective	Warfarin	635	2.3 ± 1.7	3	Restarted	NA	NA	9
Themistoclakis et al, 2010^[14]^(multicenter)	Retrospective	Warfarin	3355	ON-OAC 2.0 ± 1.3 OFF-OAC 2.3 ± 1.1	3	Restarted	0 = 60%, 1 = 27%, ≥2 = 13% (0.4†)	0 = 23%, 1 = 39%, ≥2 = 38% (1.15†)	9
Yagishita et al, 2011^[15]^(Japan)	Prospective	Warfarin	524	3.6 ± 1.1	3	Physician discretion	0 = 49%, 1 = 36%, ≥2 = 15% (0.66†)	0 = 46%, 1 = 36%, ≥2 = 18% (0.72†)	6
Saad et al, 2011^[16]^(Brazil)	Prospective	Warfarin	327	3.8 ± 1.4	3	Restarted	NA	NA	8
Hussein et al, 2011^[17]^(USA)	Prospective	Warfarin	831	4.6*	12	Unclear	NA	NA	9
Hunter et al, 2011^[18]^(UK/Australia)	Prospective	Warfarin	1273	3.1	3	Unclear	0.7 ± 0.9	0.9 ± 0.9	5
Guiot et al, 2012^[19]^(USA)	Retrospective	Warfarin	766	5.0*	3	Unclear	NA	NA	8
Winkle et al, 2013^[20]^(USA)	Prospective	Warfarin/NOAC	108	2.8 ± 1.6	7	Unclear	NA	NA	7
Gaita et al, 2014^[21]^(Italy)	Prospective	Warfarin	766	5.0*	3	Restarted	≤1 = 91.8%, ≥2 = 8.2% (1.06†,1.3‡^,^†)	≤1 = 70.4%, ≥2 = 29.6% (1.30†,2.6‡^,^†)	8
Karasoy et al, 2015^[22]^(Denmark)	Retrospective	Warfarin	4050	3.4*	3	Unclear	NA	NA	9
Kochhauser et al, 2017^[23]^(Canada)	Retrospective	Warfarin/NOAC	398	1.7*	3	Physician discretion	0 = 53%, 1 = 40%, ≥2 = 7% (0.6†,1.08‡^,^†)	0 = 17%, 1 = 36%, ≥2 = 47% (1.5†,2.52‡^,^†)	7
Okumura et al, 2019^[24]^(Japan)	Retrospective	Warfarin/NOAC	3451	1.7*	5	Unclear	0.9 ± 0.9 1.6 ± 1.3‡	1.5 ± 1.2 2.5 ± 1.6‡	9
Hermida et al, 2020^[25]^(France)	Prospective	Warfarin/NOAC	450	2.2*	3	Unclear	0.7 ± 1.0‡	1.8 ± 1.3‡	9
Yang et al, 2020^[26]^(China)	Prospective	Warfarin/NOAC	4512	ON-OAC 1.9 ± 1.1 OFF-OAC 2.0 ± 1.2	12	Restarted	2.3 ± 1.3‡	2.7 ± 1.4‡	9
Yu et al, 2020^[27]^(China)	Prospective	Warfarin/NOAC	1491	2.3 ± 1.2	3	Physician discretion	1.5 ± 1.4‡	2.4 ± 1.7‡	9

NA = not available; NOAC = non-vitamin K antagonist oral anticoagulant; NOS = Newcastle-Ottawa score; OAC = oral anticoagulant.

* Average.

† Median.

‡ CHA_2_DS_2_-VASc score.

### 3.3. Risk of thromboembolism events in OFF-OAC and ON-OAC patients after AF ablation

**Figure 2.**
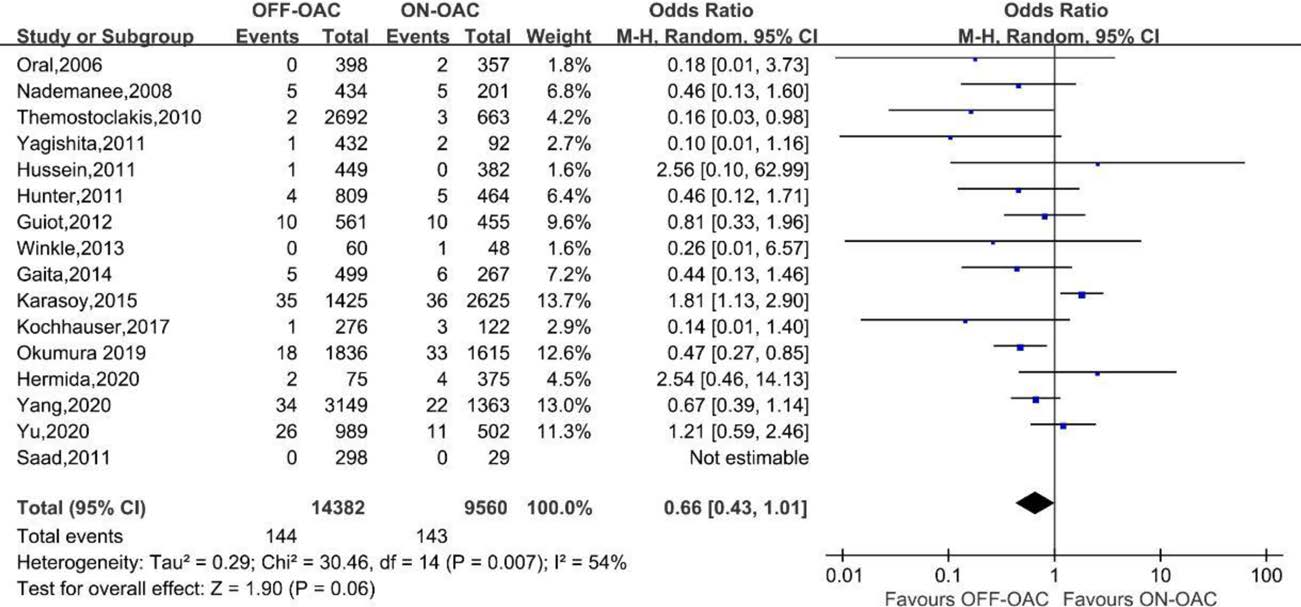
Forest plot reporting the OR for TE events in OFF-OAC and ON-OAC patients after AF ablation. AF = atrial fibrillation; OAC = oral anticoagulant; OR = odds ratio; TE = thromboembolism.

TEs were reported in 16 studies involving 23,942 patients.^[12–27]^ A study^[16]^ had zero events in both groups and the OR couldn’t be calculated. In the OFF-OAC group, 144 (1.0%) TEs occurred and 143 (1.5%) TEs occurred in the ON-OAC group. A pooled OR of 0.66 (95%CI: 0.43–1.01) was found for TEs among AF ablation patients (Fig. 2). Heterogeneity between studies was moderate (*I*^2^ = 54%, *P* = .007). Neither Begg nor Egger tests revealed any significant publication bias (*P* = .553, 0.074, respectively) (Fig. S1, Supplemental Digital Content, http://links.lww.com/MD/K187).

### 3.4. Risk of MBEs in OFF-OAC and ON-OAC patients after AF ablation

**Figure 3.**
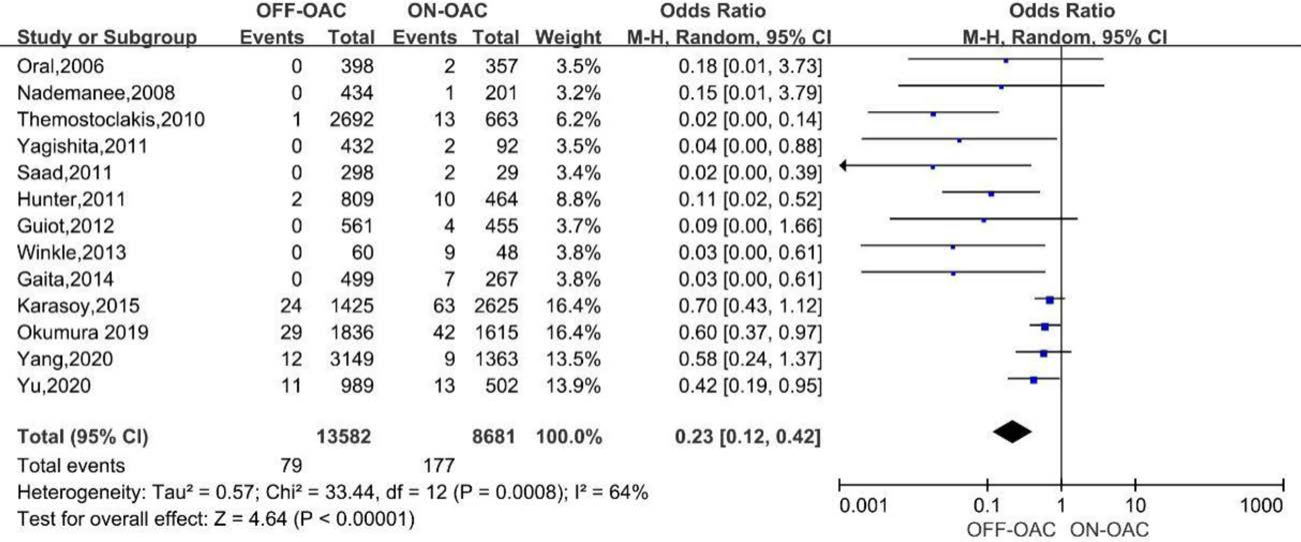
Forest plot reporting the OR for MBEs in OFF-OAC and ON-OAC patients after AF ablation. AF = atrial fibrillation; MBEs = major bleeding events; OAC= oral anticoagulant; OR = odds ratio.

A total of 13 studies including 22,263 patients reported this outcome.^[12–16,18–22,24,26,27]^ In the OFF-OAC group, 79 (0.6%) MBEs occurred and 321 (2.0%) MBEs occurred in the ON-OAC group. In patients after AF ablation, the pooled OR for MBEs was 0.23 (95%CI: 0.12–0.42) (Fig. 3). There was moderate heterogeneity between the studies (*I*^2^ = 64%, *P* = .0008). Begg test did not reveal any significant publication bias (*P* > .05).

### 3.5. 3.5. Risk of thromboembolism events in OFF-OAC and ON-OAC patients after AF ablation stratified by the risk level of thromboembolism assessed by CHA_2_DS_2_-VASc or CHADS_2_ score

**Figure 4.**
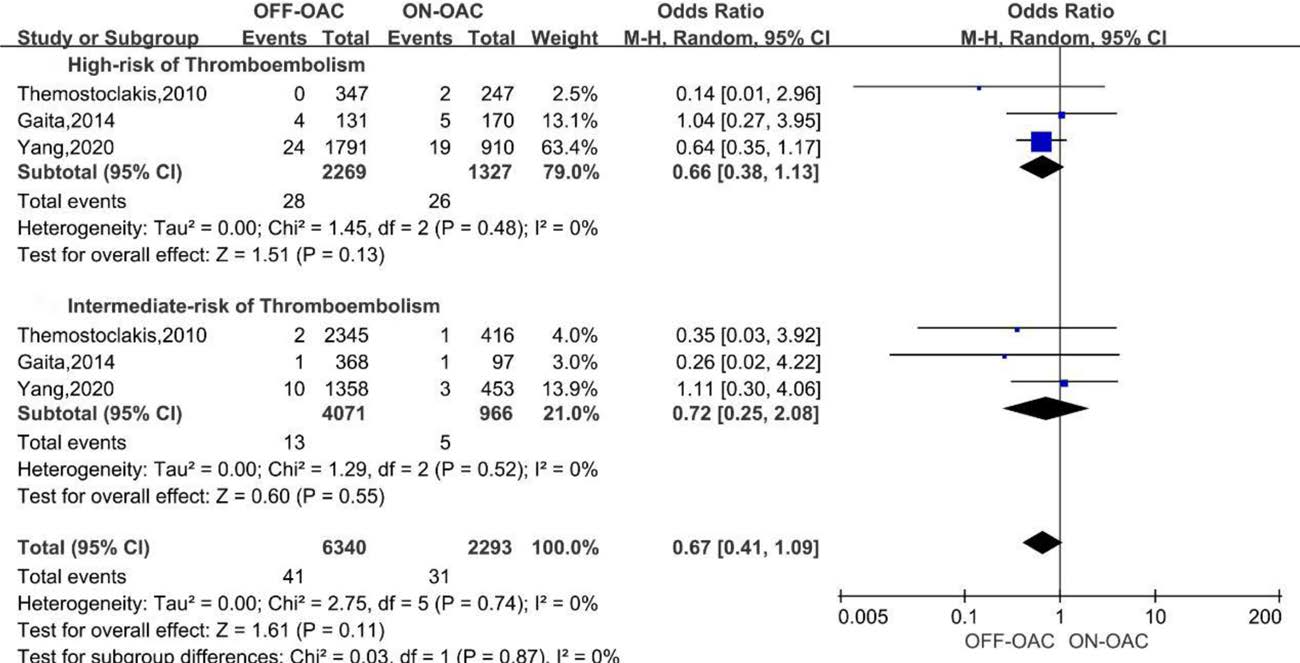
Forest plot reporting the OR for TE events in OFF-OAC and ON-OAC patients after AF ablation stratified by the risk level of thromboembolism. AF = atrial fibrillation; OA = oral anticoagulant; OR = odds ratio; TE = thromboembolism.

Two studies^[21,26]^ reported the number of TEs in both groups stratified by CHA_2_DS_2_-VASc and one study^[14]^ reported the number of TEs in both groups stratified by CHADS_2_ score. CHA_2_DS_2_-VASc or CHADS_2_ ≥ 2 was assessed as a high risk of thromboembolism and CHA_2_DS_2_-VASc or CHADS_2_ < 2 was assessed as an intermediate risk. In the group with a high risk of thromboembolism, 2269 patients were OFF-OAC and 1327 were ON-OAC, and in the group with an intermediate risk of thromboembolism, 4071 patients were OFF-OAC and 966 were ON-OAC. The pooled OR for TEs in high-risk was 0.66 (95%CI: 0.38–1.13), while 0.72 (95%CI: 0.25–2.08) for TEs in intermediate-risk (Fig. 4). Both groups of studies did not show any heterogeneity (*I*^2^ = 0 for both, *P* = .48 and 0.52, respectively). A publication bias analysis was not performed because of the limited number of stratified analyses. The stratified analysis did not show significant differences between the OFF-OAC and ON-OAC groups at both risk levels.

### 3.6. Sensitive analysis and subgroup analyses for thromboembolism events

**Figure 5.**
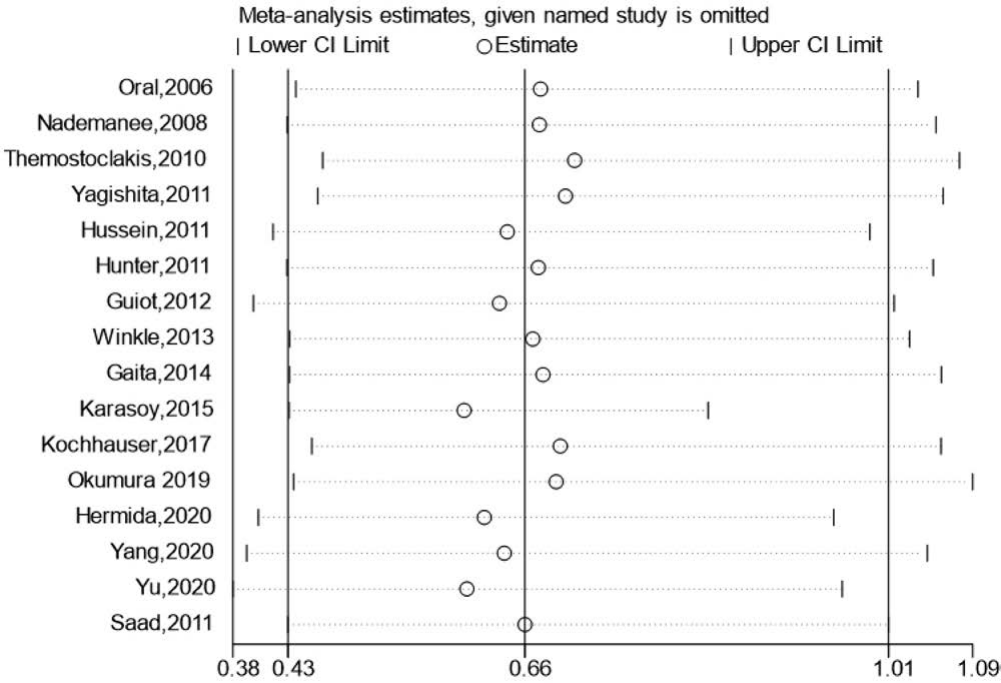
Result of sensitive analysis for TE events. Each row presents the overall combined OR and CI after exclusion of every single study. CI = confidence interval; OR = odds ratio.

**Figure 6.**
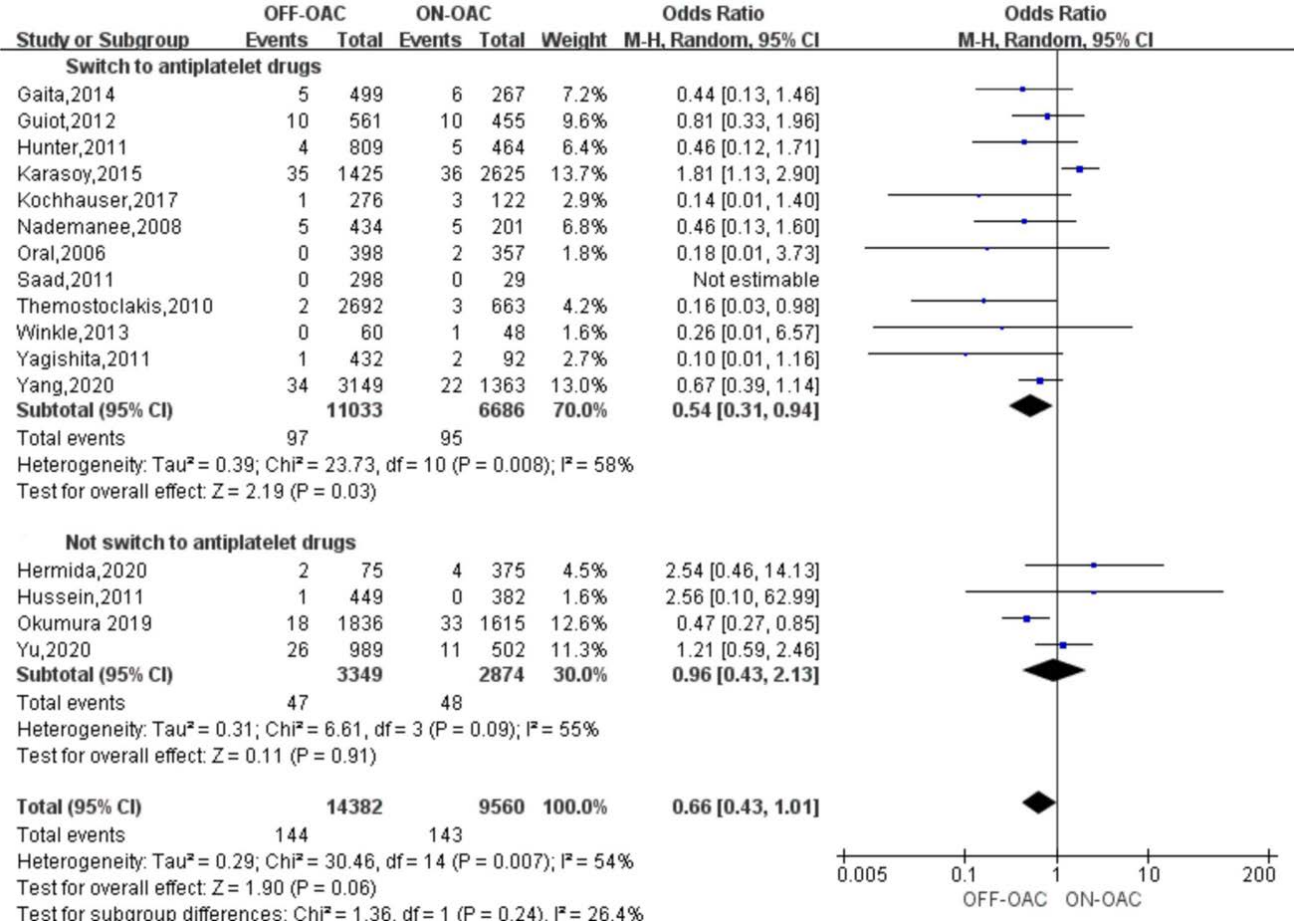
Forest plot reporting the OR for TE events in OFF-OAC and ON-OAC patients after AF ablation according to whether switch to antiplatelet drugs. AF = atrial fibrillation; OAC = oral anticoagulants; OR = odds ratio; TE = thromboembolism.

Sensitive analysis was performed by the exclusion of a single study one by one and the combined ORs did not significantly change with the exclusion of any single study. Figure 5 presents the result of the sensitivity analysis. Subgroup analyses for thromboembolism events were performed according to the study design (prospective vs retrospective), study region (Asian vs non-Asian), OAC type (warfarin vs warfarin or NOACs), whether switch to antiplatelet drugs (switch to antiplatelet drugs vs not switch to antiplatelet drugs), and follow-up duration (<3 years vs ≥3 years). In the subgroup of whether switching to antiplatelet drugs, the pooled ORs for TEs in switching to antiplatelet drugs was 0.54 (95%CI: 0.31–0.94), while 0.96 (95%CI: 0.43–2.13) for TEs in not switching to antiplatelet drugs (Fig. 6). The pooled ORs are significantly different in the subgroups according to follow-up duration (Fig. S2, Supplemental Digital Content, http://links.lww.com/MD/K188). No significant difference in other subgroups (Figs. S3–S5, Supplemental Digital Content, http://links.lww.com/MD/K189, http://links.lww.com/MD/K190, http://links.lww.com/MD/K191). Table 2 presents the overall results of the subgroup analyses.

**Table 2:** Subgroup analyses based on characteristics of included studies for thromboembolism in patients after AF ablation.

Subgroup	Variable	Number of studies	OR (95%CI)
Study design	Prospective	11	0.69 (0.46,1.03)
	Retrospective	5	0.60 (0.25,1.43)
Region	Asian	4	0.64 (0.37,1.11)
	Non-Asian	12	0.65 (0.35,1.20)
OAC type	Warfarin	10	0.57 (0.28,1.14)
	Warfarin or NOACs	6	0.70 (0.42,1.17)
Whether switch to antiplatelet drugs	Switch to antiplatelet drugs	12	0.54 (0.31,0.94)
	Not switch to antiplatelet drugs	4	0.96 (0.43,2.13)
Follow-up duration	<3 years	9	0.60 (0.38,0.95)
	≥3 years	7	0.75 (0.35,1.62)

AF = atrial fibrillation; CI = confidence interval; OAC = oral anticoagulant; OR = odds ratio; NOACs = non-vitamin K antagonist oral anticoagulants.

### 3.7. Subgroup analysis for MBEs

**Figure 7.**
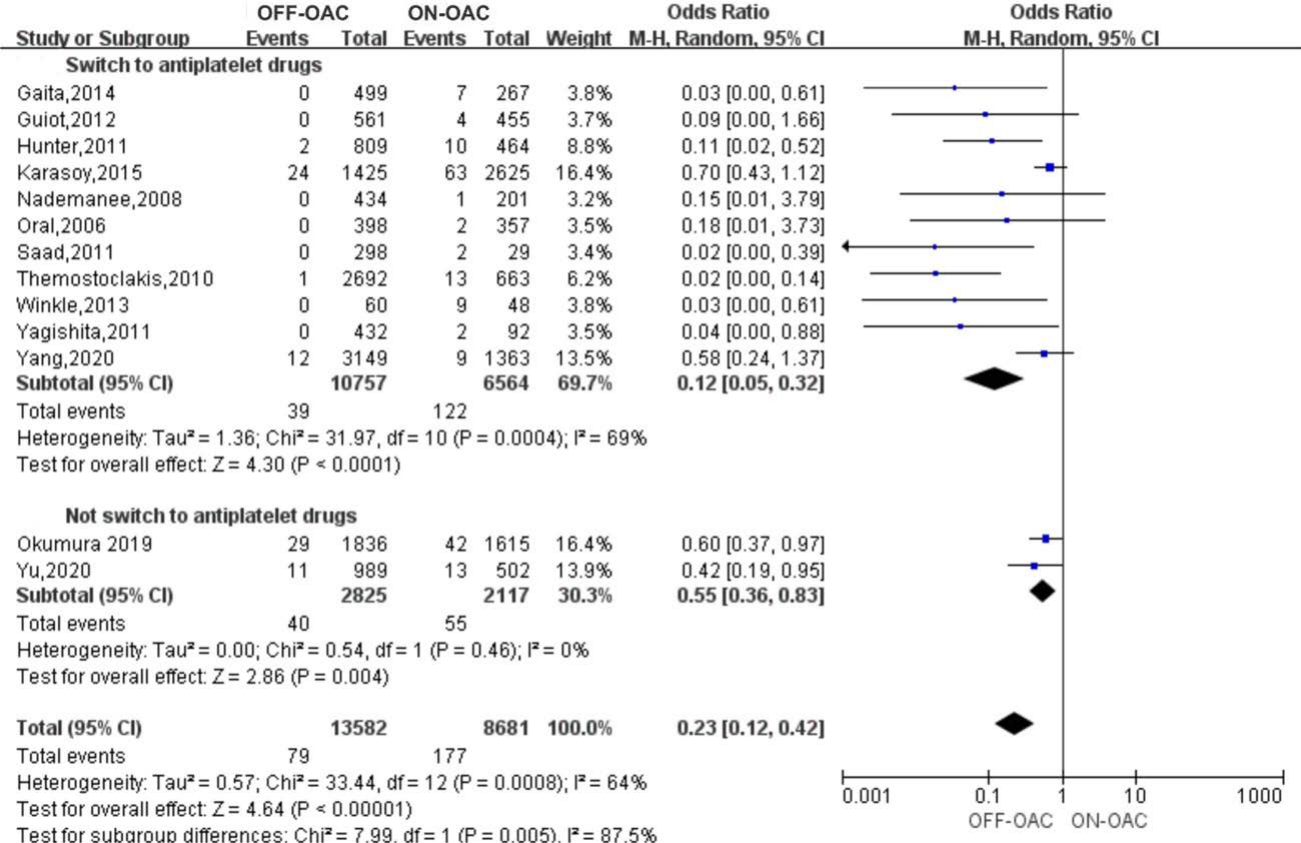
Forest plot reporting the OR for MBEs in OFF-OAC and ON-OAC patients after AF ablation according to whether switch to antiplatelet drugs. AF = atrial fibrillation; MBEs = major bleeding events; OAC = oral anticoagulants; OR = odds ratio.

Subgroup analyses for MBEs were performed according to the study design (prospective vs retrospective), study region (Asian vs non-Asian), OAC type (warfarin vs warfarin or NOACs), whether switch to antiplatelet drugs (switch to antiplatelet drugs vs not switch to antiplatelet drugs), and follow-up duration (<3 years vs ≥3 years). In the subgroup of whether switching to antiplatelet drugs, the pooled ORs for MBEs in switching to antiplatelet drugs was 0.12 (95%CI: 0.05–0.32), while 0.55 (95%CI: 0.36–0.83) for MBEs in not switching to antiplatelet drugs (Fig. 7). The pooled ORs suggested a significant reduction in the incidences of MBEs in all subgroups (Figs. S6–S9, Supplemental Digital Content, http://links.lww.com/MD/K192, http://links.lww.com/MD/K193, http://links.lww.com/MD/K194, http://links.lww.com/MD/K195). Table 3 presents the results of the subgroup analyses.

**Table 3:** Subgroup analyses based on characteristics of included studies for major bleeding events in patients after AF ablation.

Subgroup	Variable	Number of studies	OR (95%CI)
Study design	Prospective	9	0.17 (0.07,0.39)
	Retrospective	4	0.33 (0.12,0.85)
Region	Asian	4	0.53 (0.35,0.79)
	Non-Asian	9	0.09 (0.02,0.32)
OAC type	Warfarin	9	0.09 (0.03,0.32)
	Warfarin or NOACs	4	0.50 (0.30,0.82)
Whether switch to antiplatelet drugs	Switch to antiplatelet drugs	11	0.12 (0.05,0.32)
	Not switch to antiplatelet drugs	2	0.55 (0.36,0.83)
Follow-up duration	<3 years	7	0.28 (0.13,0.62)
	≥3 years	6	0.23 (0.02,0.47)

AF = atrial fibrillation; CI = confidence interval; OAC = oral anticoagulant; OR = odds ratio; NOACs = non-vitamin K antagonist oral anticoagulants.

## 4. Discussion

The current guideline recommended OACs should be maintained for at least 2 months after AF ablation and an individual patient’s risk of stroke should be taken into account when deciding whether to continue OACs.^[4]^ However, there is a gap between the guideline and clinical practice regarding the status of anticoagulation in AF patients. A recent population-based study showed that only 74% of patients with atrial fibrillation could take OACs as prescribed.^[28]^ Real-world evidence indicated that 15% of patients received inappropriate NOACs dosage^[29]^ and in elderly patients, this proportion could be as high as 30%.^[30]^ Intensive targeted education and AF monitoring may improve anticoagulation status.^[31]^ However, if selected AF patients can discontinue OACs safely, it would facilitate the management of atrial fibrillation. The evidence evaluating this topic was limited to observational studies and this meta-analysis aimed to determine how to balance the risk of thromboembolism and major bleeding. In our meta-analysis, we observed a significant reduction in MBEs with the discontinuation of OACs after successful catheter ablation of AF, and TE events did not differ significantly between the 2 groups. Previous meta-analyses have found similar results.^[32,33]^ Compared with the published articles, we conducted stratified analysis by the risk of stroke to determine the exact incidence rate of TE in different risk level groups, and we included all cohort studies meeting inclusion criteria to expand the sample while eliminating the study of patients who discontinued OACs within 3 months after ablation. All we did can make the results more stable and credible to some extent.

We found no significant difference in TE events between OFF-OAC and ON-OAC groups. There is homogeneity in patients included in our analysis of main characteristics. All ablation procedures contain pulmonary vein isolation (PVI). All patients continued antiarrhythmic drug therapy and OACs at least 3 months after ablation. Our research emphasizes the importance of the blanking period. AF patients after ablation can be susceptible to thrombosis due to activation of the pro-coagulant cascade followed by the endocardial pro-inflammatory activation and hemodynamics alteration after restoring sinus rhythm^[34]^ and this state can last for several weeks. For this reason, following a successful ablation, it is recommended that OACs continued for at least 2 months.^[4]^ An analysis of retrospective cohort data conducted by Själander et al^[35]^ showed that patients who discontinued warfarin after PVI with a CHA_2_DS_2_-VASc score of 2 or more had a higher risk of ischemic stroke and there was no significant difference in the rate of stroke between patients with or without warfarin in patients with a CHA_2_DS_2_-VASc score of 0 or 1. However, in this study, some of the patients discontinued OACs in the first 3 months, which may be the cause of the difference in our results. The results of our pooled analysis suggested that in AF patients who have had their ablation successfully, OACs could be discontinued safely. There is a possibility that the benefit is related to sinus rhythm maintenance. During a long-term follow-up of the EAST-AFNET 4 trial, early rhythm control therapy reduced cardiovascular and stroke-related mortality compared with usual care.^[7]^ Potential mechanisms of the lower stroke risks may be attributed to the reduction of AF burden and reverse left atrium remodeling.^[36]^

Our results should be interpreted and applied with caution. The results of included studies were moderately heterogeneous, which can be attributed to differences in article design and unobserved confounders. Due to the lack of single-patient data, patient characteristics cannot be adjusted and further analyzed, which can also be potential confounders. There can be an interaction between CHA_2_DS_2_-VASc or CHADS_2_ scores and the decision of anticoagulation. Patients who discontinued OACs always had lower CHADS_2_ or CHA_2_DS_2_-VASc scores than patients who continued OACs, thus we conducted a stratified analysis in patients stratified by TE risk scores. Only 3 studies supplied an exact number of events stratified by TE risk scores, and the results supported that discontinuing OACs could be safe even when patients have a high risk of TE. However, in most studies, lower TE risk for OFF-OAC groups reflected selection bias that the lower TE rates appeared in a highly selected population of post-ablation patients, which weakened the reliability of the results. Meanwhile, AF recurrence and reinitiation of OACs were not properly assessed in most of the studies, which might critically affect the results.

We found a significant reduction in MBEs in OFF-OAC groups, although we were unable to assess bleeding risks in the total sample population because of the lack of data in original articles. However, the estimated bleeding risk assessed by HAS-BLED score or other tools is not recommended to guide the decision to use OACs for stroke prevention in the absence of absolute contraindications to OACs,^[4]^ thus the lack of this section of data would not adversely affect our results greatly. The reduction of MBEs also may result from selection bias. Despite the discovery of reduction in MBEs with discontinuation of OACs, it doesn’t mean that we encourage discontinuation of OACs due to the risk of bleeding, and it just means that discontinuation of OACs in selective AF patients after ablation may reduce the risk of bleeding without increasing risk of thromboembolism and points that we should focus on potentially modifiable risk factors of bleeding.

Of note, we conducted subgroup analyses to evaluate possible factors that may impact the results. The pooled ORs of the subgroup analyses for MBEs suggested a significant reduction in the incidences of MBEs in all subgroups and we found significant differences in the incidences of TEs in the subgroups according to whether switching to antiplatelet drugs and follow-up duration. It is common to stop OACs and switch to antiplatelet drugs without the indication of antiplatelet drugs in the AF patients after ablation, and the reduction of the incidences of TEs in the patients who switched to antiplatelet drugs met our expectations. The evidence of the effect of antiplatelet drugs in the stroke prevention mainly came from patients with atrial fibrillation who had not accepted catheter ablation,^[37,38]^ and cardiovascular society guidelines recommended against using antiplatelet therapy alone in patients with AF just for stroke prevention regardless of stroke risk.^[39]^ However, patients who accepted ablation may have different characteristics from those who have not accepted ablation and the prevalence of TEs is very low in the patients who accepted ablation compared to those who have not.^[40]^ Catheter ablation led to a new era of rhythm control. Whether can catheter ablation lead to a new era of anticoagulation therapy? Further investigation and clarification of the clinical characteristics of AF patients after ablation are important. Our results suggested the safety of discontinuation of OACs even in patients who switched to antiplatelet drugs with a reduction of incidences of major bleeding. However, our analysis inevitably had all associated inherent bias as it is based on observational studies. A lack of randomization may result in an important selection bias. Well-designed RCTs are required to confirm our findings. Otherwise, a significant difference was found in the subgroup according to follow-up duration. We chose 3 years as a cutoff value with experience, however, 5 years was the longest follow-up period of included studies. Whether our findings can be extended to a very-long follow-up duration needs further exploration. The difference in the subgroup may only reflect the trend of increased detection rate of incidences with the extension of the follow-up.

We found no difference in other subgroup analyses of TEs. However, we still need to pay attention to the potential bias in subgroups. Some biases were obvious directional which reflected the development and progress of the drugs and recognition of AF anticoagulation. Firstly, in early studies, vitamin K antagonist anticoagulant (VKA) was the only OACs, and in recent studies, a large proportion of patients applied NOACs. Some high-quality RCTs and meta-analyses have confirmed that NOACs are non-inferior or superior to VKA in stroke prevention with a reduced intracranial hemorrhage risk.^[38,41]^ Since NOACs have a rapid onset-offset of action and are not required to monitor the international normalized ratio frequently, NOACs are convenient for both physicians and patients so that patients can achieve good adherence.^[42]^ Current guidelines recommended that VKA should be switched to NOACs in patients with time in therapeutic range (TTR) < 70% and a growing number of patients choose to apply NOACs at the beginning.^[4,43]^ Secondly, some studies may underestimate the probability of AF recurrence for a lack of continuous monitoring systems, which can overestimate the risk of TE in ON-OAC patients. Otherwise, the reinitialization of OACs after AF recurrence is different among included studies, which may affect the results potentially.

The major cardiovascular society guidelines seem to tend to recommend the approach that has been proved also effective in AF patients without ablation conservatively. 2014 AHA/ACC/HRS Guideline for the Management of Patients With Atrial Fibrillation emphasized that AF catheter ablation to restore sinus rhythm should not be performed with the only purpose of obviating the need for anticoagulation.^[44]^ 2017 HRS/EHRA/ECAS/APHRS/SOLAECE expert consensus statement on catheter and surgical ablation of atrial fibrillation also recommend that decisions to discontinue OACs should be based on individual patients’ stroke risk regardless of the outcome of catheter ablation.^[45]^ The main concern of major cardiovascular society guidelines probably was the asymptomatic recurrence of atrial fibrillation. Asymptomatic episodes of AF could be detected in patients with an apparently successful ablation procedure during long-term follow-up. According to a meta-analysis of 13 studies, only 59% of AF patients with a single PVI experienced no arrhythmia after 5 years.^[46]^ 40% of patients with AF could be asymptomatic and asymptomatic patients presented higher 1-year mortality compared with symptomatic patients for lower use of anticoagulation.^[47]^ However, we need not be pessimistic about it. The publication of high-quality RCTs and rapid development of AF catheter ablation as well as diagnostic possibilities promote the update of guidelines. The presentation of “4S AF scheme” and “ABC pathway” enhance the management of AF.^[4]^ Patients with persistent AF can also benefit from targeted therapy for underlying conditions in RACE 3.^[48]^ Technologies of AF detection are also evolving rapidly, which can identify patients with asymptomatic AF very well.^[49]^ More and more smart and wearable devices are applied to AF patients with good accuracy, and AF burden can be detected by these devices rather than AF recurrence to assist physicians in making decisions on stroke prevention strategies. Such shreds of evidence ensure the benefit of catheter ablation. The 2020 ESC AF guidelines also highlight the focus on patient involvement to achieve optimal outcomes.^[4]^ We should consider the incorporation of patient values to achieve shared decision-making. Our results can give a choice for patients who are able and willing to assess their heart rhythm and identify asymptomatic episodes of AF. In addition, when to discontinue OACs can be an important question and needs further investigation.

Clinical evidence is also being accumulated constantly. The results of the ongoing ODln-AF trial (NCT02067182) and OCEAN trial (NCT02168829) will provide specific evidence for answering whether we can stop OACs in selective AF patients after successful ablation safely and whether we need to convert OACs to antiplatelet drugs after stopping OACs. We look forward to the results of trials updating clinical evidence leading to guidelines better executed and reducing the translational gap between guidelines and clinical practice.

## 5. Conclusion

The results of our meta-analysis suggest that discontinuing OACs after successful AF ablation can be safe. Discontinuing OACs does not cause an increase in TEs while reducing MBE risk. Switching to antiplatelet drugs after discontinuation of OACs in patients after ablation can reduce the incidences of TEs with the reduction of the incidences of major bleeding. However, due to the heterogeneity of included studies and the limitation of study designs, our results should be interpreted with caution. We need to confirm our findings with large-scale and well-designed RCTs.

## Author contributions

**Conceptualization:** Xiangyu Wang, Minghua Li, Zhiguo Zhang.

**Data curation:** Xiangyu Wang, Minghua Li, Xishu Wang.

**Formal analysis:** Xiangyu Wang, Minghua Li.

**Investigation:** Xiangyu Wang, Minghua Li.

**Methodology:** Xiangyu Wang, Zhiguo Zhang.

**Resources:** Xiangyu Wang.

**Software:** Xiangyu Wang.

**Supervision:** Xiangyu Wang.

**Visualization:** Xiangyu Wang.

**Writing – original draft:** Xiangyu Wang, Xishu Wang.

**Writing – review & editing:** Xiangyu Wang, Zhiguo Zhang.

## Supplementary Material




















