# Supplementary material for: It can be safe to discontinue oral anticoagulants after successful atrial fibrillation ablation: A systematic review and meta-analysis of cohort studies
Source: Medicine (Baltimore). 2023 Oct 20;102(42):e35518. doi: 10.1097/MD.0000000000035518 (PMC10589570; doi:10.1097/MD.0000000000035518)
Supplement: Supplementary file 1 [file medi-102-e35518-s001.pdf]

Supplementary Table 1. Literature Search strategy

**Literature Search strategy via PubMed**<br><br>#1 atrial fibrillation[Title/Abstract]<br>#2 atrial fibrillation[MeSH Terms]<br>#3 (atrial[Title/Abstract] OR atrium[Title/Abstract] OR auricular) fibrillat*[Title/Abstract]<br>#4 ablation[Title/Abstract]<br>#5 catheter ablation[MeSH Terms]<br>#6 catheter ablat*[Title/Abstract]<br>#7 percutaneous catheter*[Title/Abstract]<br>#8 anticoagula*[Title/Abstract]<br>#9 (dabigatran[Title/Abstract]) or (apixaban[Title/Abstract]) or (rivaroxaban[Title/Abstract]) or (edoxaban[Title/Abstract])<br>#10 vitamin K antagon*[Title/Abstract]<br>#11 vitamin k inhibitor*[Title/Abstract]<br>#12 vka[Title/Abstract]<br>#13 antivitamin k[Title/Abstract]<br>#14 warfarin[Title/Abstract]<br>#15 non-vitamin K antagonist oral anticoagulant[Title/Abstract]<br>#16 direct oral anticoagulant[Title/Abstract]<br>#17 (NOAC[Title/Abstract]) OR (DOAC[Title/Abstract])<br>#18 #1 or #2 or #3<br>#19 #4 or #5 or #6 or #7<br>#20 #8 or #9 or #10 or #11 or #12 or #13 or #14 or #15 or #16 or #17<br>#21 #18 and #19 and #20<br>#22 animals[mh] NOT humans[mh]<br>#23 #21 NOT #22<br>#24 cohort studies[mesh:noexp] or longitudinal studies[mesh:noexp] or follow-up studies[mesh:noexp] pr prospective studies[mesh:noexp] or retrospective studies[mesh:noexp] or cohort[tiab] or longitudinal[tiab] or prospective[tiab] or retrospective[tiab]<br>#25 #23 and #24	**2131**
**Literature Search strategy via Embase**<br><br>#1 'atrial fibrillation'/exp OR 'atrial fibrillation'<br>#2 ablation:ti,ab,kw<br>#3 #1 and #2<br>#4 dabigatran:ti,ab,kw OR apixaban:ti,ab,kw OR rivaroxaban:ti,ab,kw OR edoxaban:ti,ab,kw OR 'vitamin k antagon*':ti,ab,kw OR warfarin:ti,ab,kw OR 'non-vitamin k antagonist oral anticoagulant':ti,ab,kw OR 'direct oral anticoagulant':ti,ab,kw OR noac:ti,ab,kw OR doac:ti,ab,kw<br>#5 #3 and #4	**1733**

Literature Search strategy via Web of Science<br><br>#1 TS=(atrial fibrillation)<br>#2 TS=(ablation)<br>#3 #1 and #2<br>#4 ((((TS=(anticoagula*)) OR TS=(dabigatran)) OR TS=(apixaban)) OR TS=(rivaroxaban)) OR TS=(edoxaban)<br>#5 (TS=(vitamin K antagon*)) OR TS=(warfarin)<br>#6 (((TS=(non-vitamin K antagonist oral anticoagulant)) OR TS=(direct oral anticoagulant)) OR TS=(NOAC)) OR TS=(DOAC)<br>#7 #4 or #5 or #6<br>#8 TS=(cohort)<br>#9 #3 and #7 and #8	203
