# Supplementary figures and images for: It can be safe to discontinue oral anticoagulants after successful atrial fibrillation ablation: A systematic review and meta-analysis of cohort studies
Source: Medicine (Baltimore). 2023 Oct 20;102(42):e35518. doi: 10.1097/MD.0000000000035518 (PMC10589570; doi:10.1097/MD.0000000000035518)

## Slide 1
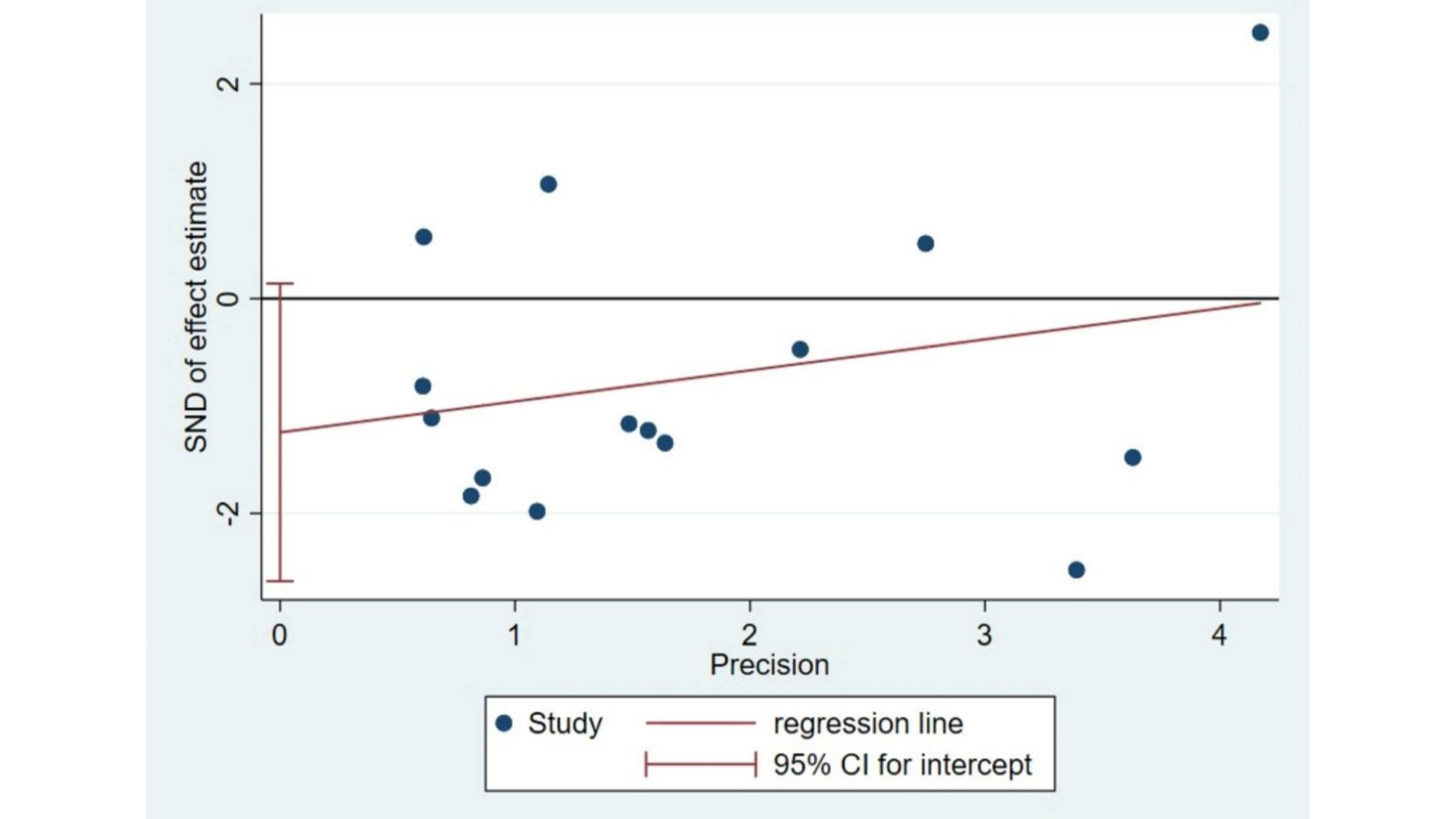

Supplement: Supplementary file 2 [file medi-102-e35518-s002.pptx]

## Slide 1
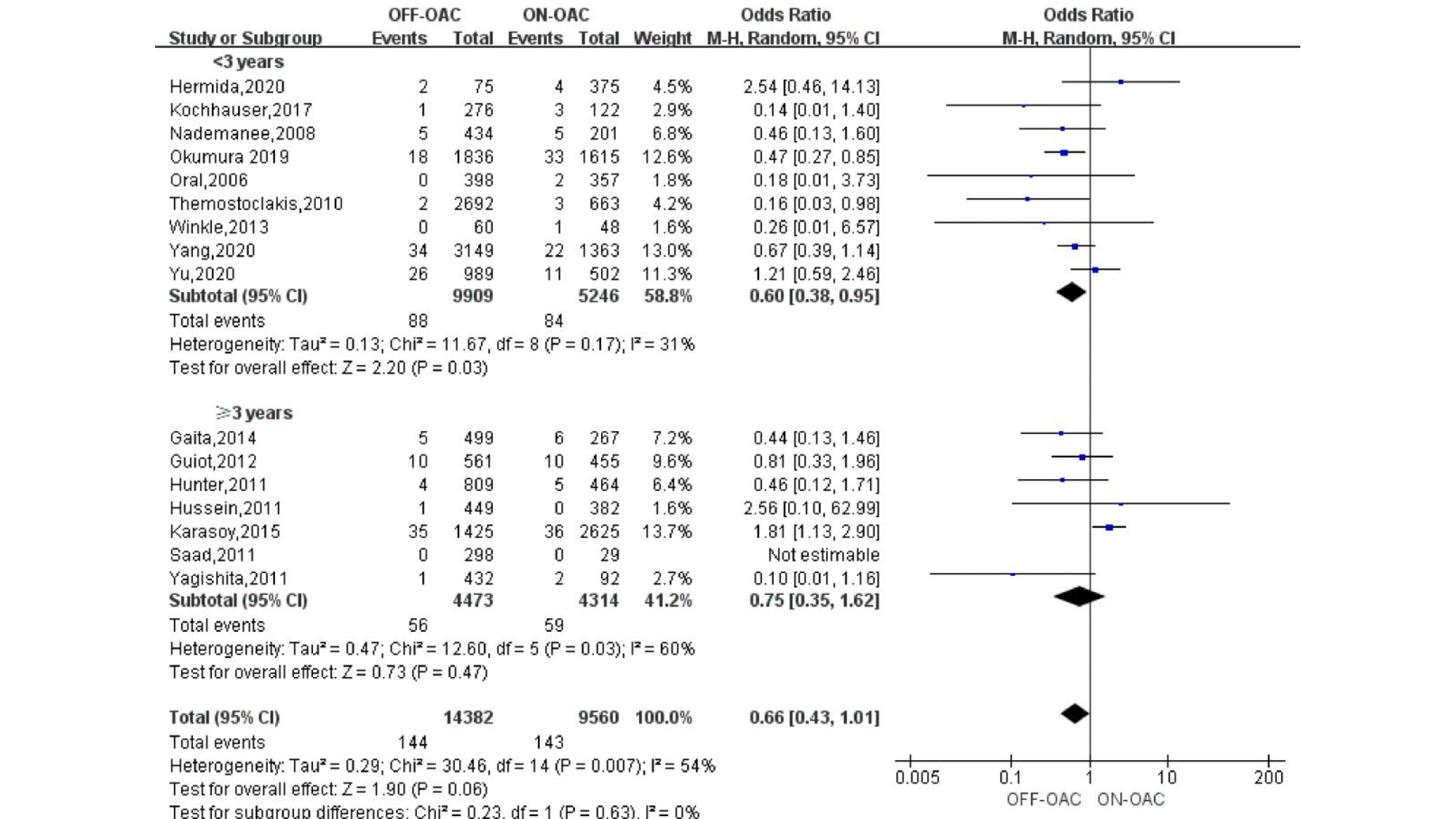

Supplement: Supplementary file 3 [file medi-102-e35518-s003.pptx]

## Slide 1
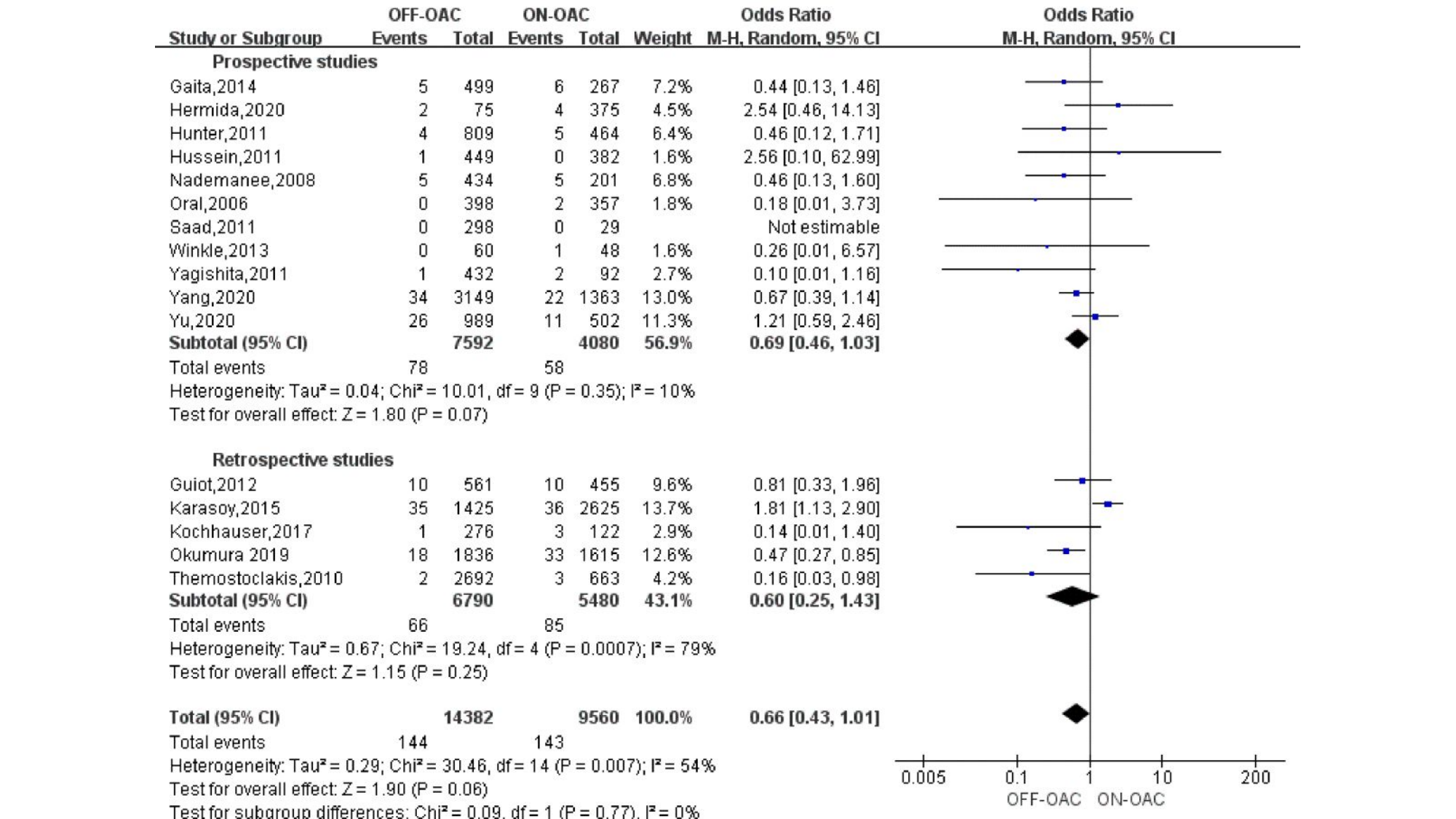

Supplement: Supplementary file 4 [file medi-102-e35518-s004.pptx]

## Slide 1
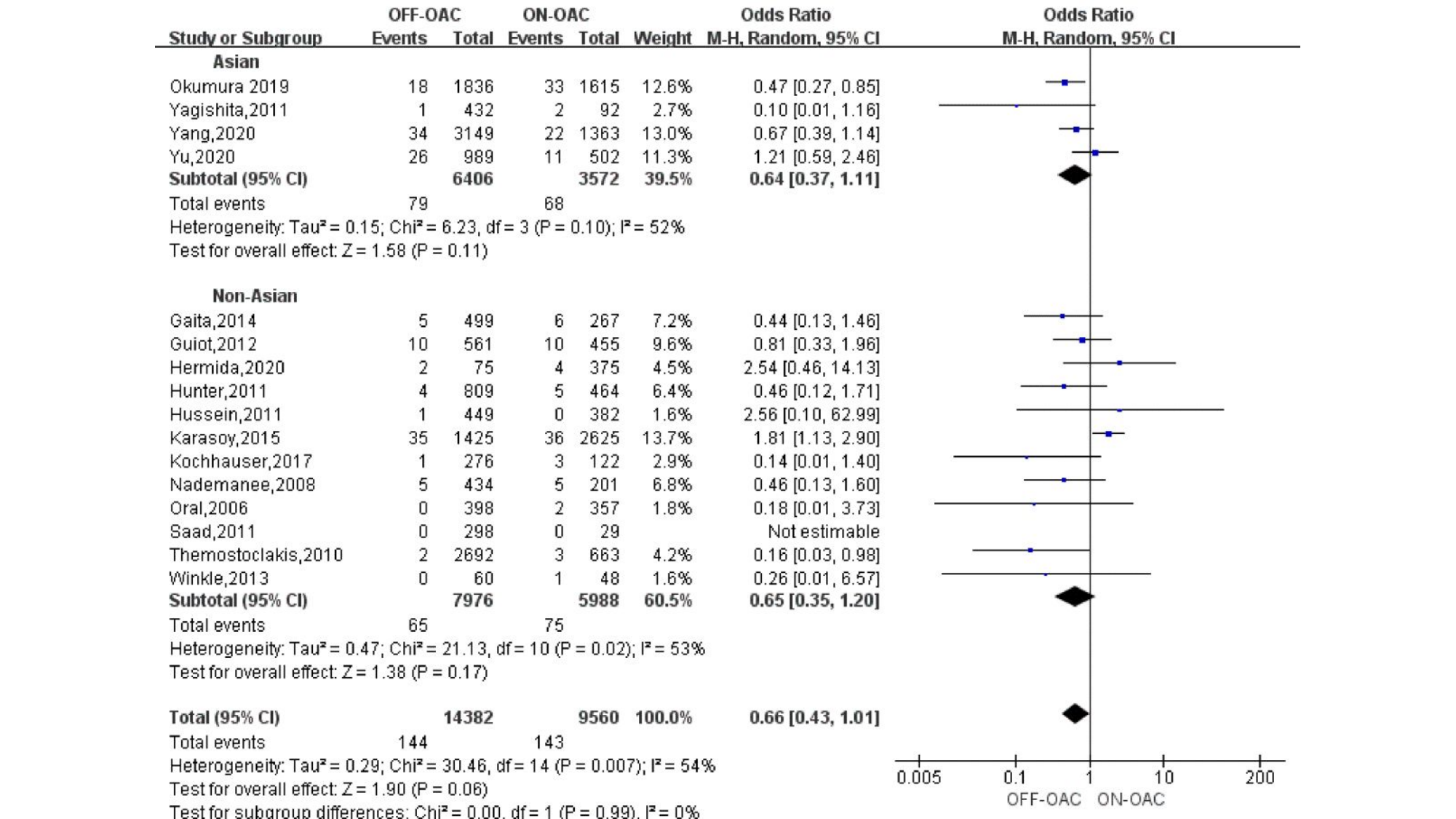

Supplement: Supplementary file 5 [file medi-102-e35518-s005.pptx]

## Slide 1
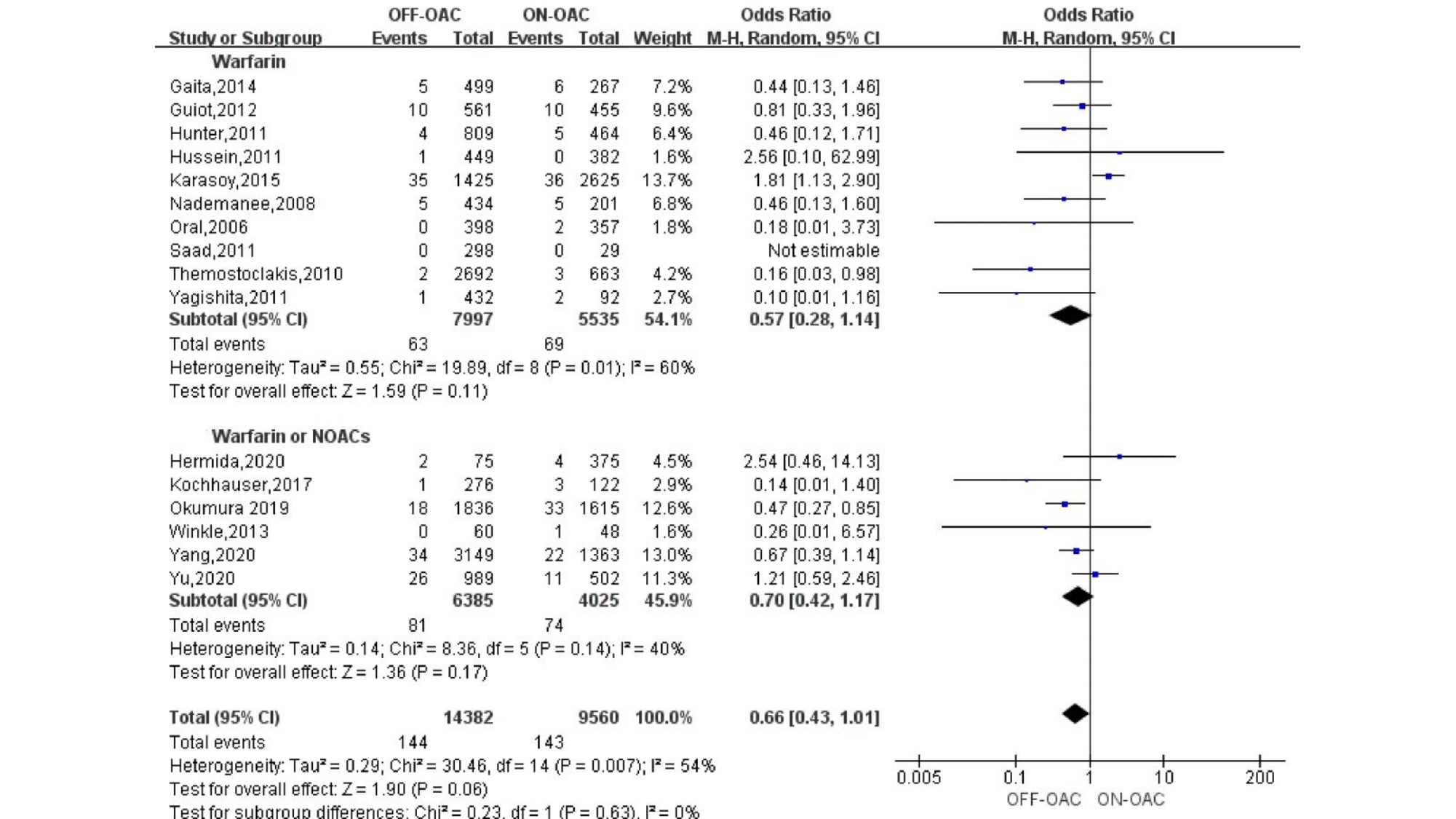

Supplement: Supplementary file 6 [file medi-102-e35518-s006.pptx]

## Slide 1
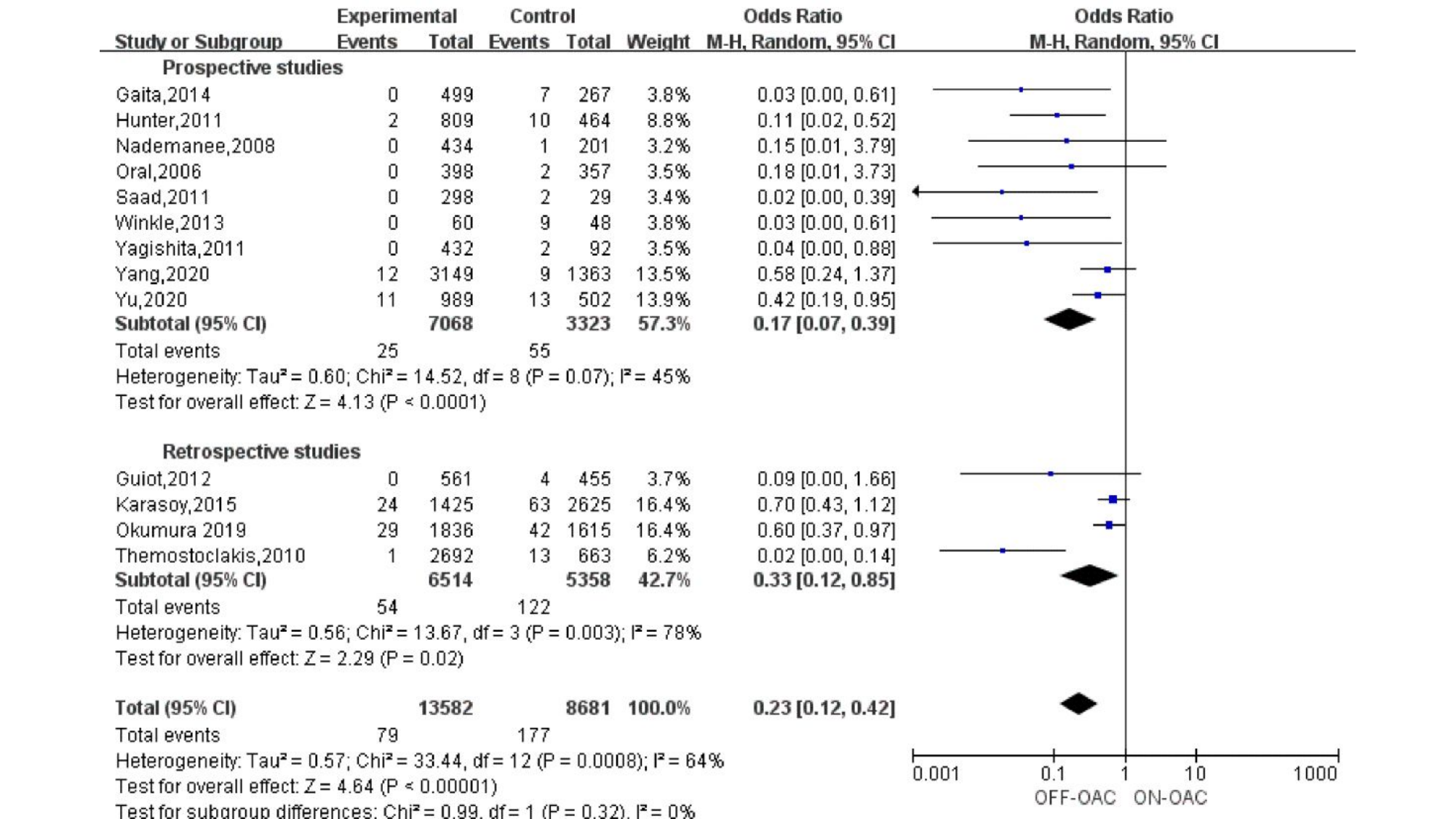

Supplement: Supplementary file 7 [file medi-102-e35518-s007.pptx]

## Slide 1
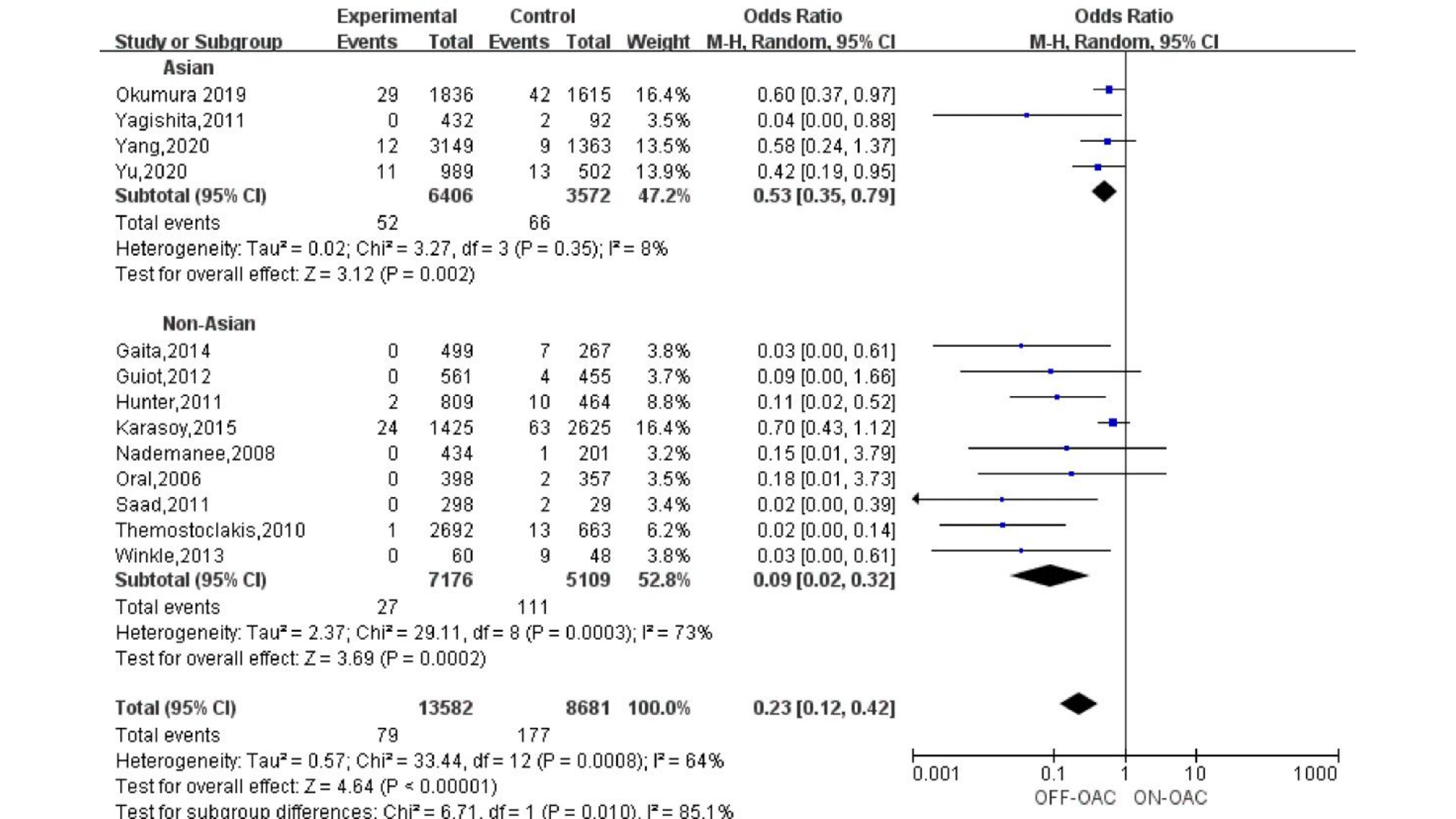

Supplement: Supplementary file 8 [file medi-102-e35518-s008.pptx]

## Slide 1
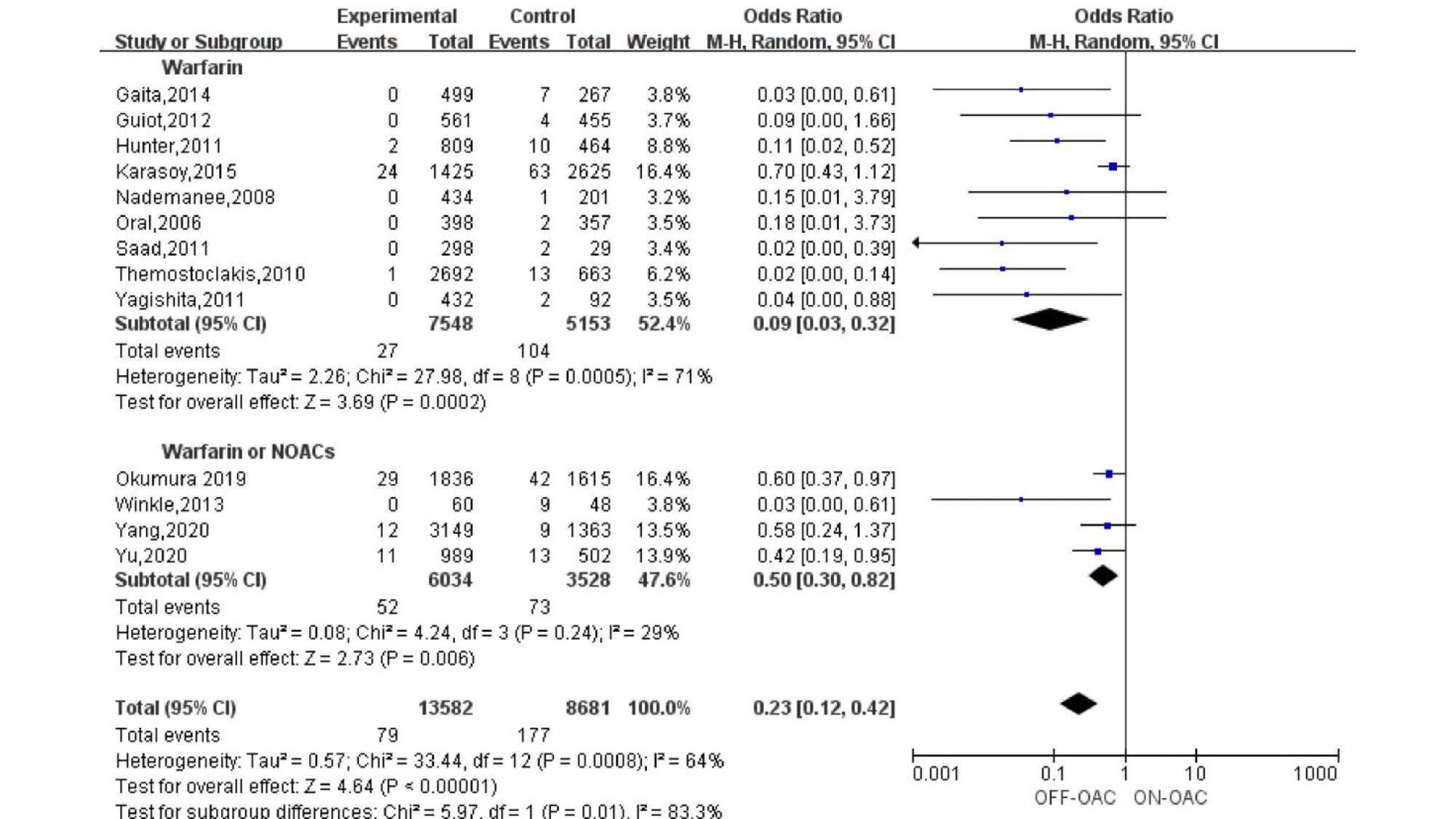

Supplement: Supplementary file 9 [file medi-102-e35518-s009.pptx]

## Slide 1
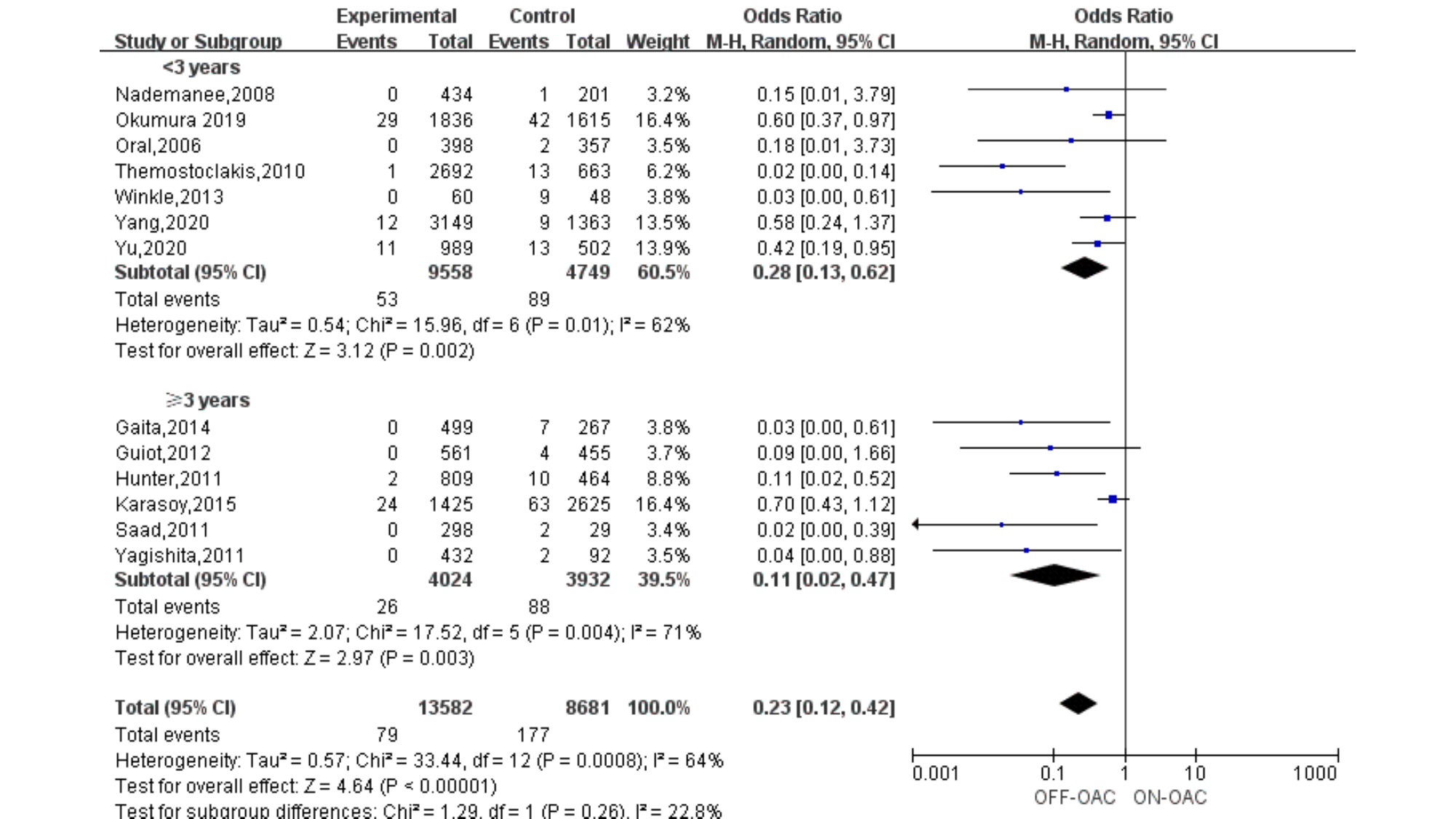

Supplement: Supplementary file 10 [file medi-102-e35518-s010.pptx]
